# PHYSICAL FRAILTY AND ITS ASSOCIATION WITH COGNITION: THE PREDICTIVE VALUE OF A SYNDROME BEYOND ITS COMPONENT PARTS

**DOI:** 10.1093/geroni/igz038.377

**Published:** 2019-11-08

**Authors:** Nadia M Chu, Karen Bandeen-Roche, A Richey Sharrett, Michelle C Carlson, Qianli Xue, Alden Gross

**Affiliations:** 1 Department of Surgery, Johns Hopkins School of Medicine, Baltimore, Maryland, United States; 2 Johns Hopkins Bloomberg School of Public Health, Baltimore, Maryland, United States; 3 Johns Hopkins University, Baltimore, Maryland, United States; 4 Department of Medicine, School of Medicine, Johns Hopkins University, Baltimore, Maryland, United States; 5 Johns Hopkins University Bloomberg School of Public Health, Baltimore, Maryland, United States

## Abstract

The extent to which frailty (PFP) affects cognitive performance and change beyond that expected from its component parts is uncertain. Leveraging NHATS, a nationally-representative cohort of U.S. Medicare beneficiaries, we quantified associations between each PFP criterion and global and domain-specific cognitive level and change (memory: immediate/delayed word-list test, executive function: clock drawing test (CDT), orientation: date, time, president-vice-resident naming), using adjusted mixed effects models with random slopes (time) and intercepts (person). We tested whether presence of frailty was associated with excess cognitive vulnerability (synergistic/excess effects, Cohen’s d) above and beyond those found for its criteria by adding an interaction term between each PFP criterion and frailty. Among 7,439 community-dwelling older adults (mean age=75.2 years) followed for a weighted mean of 3.2 years (SE= 0.03), 14.1% were frail. The most prevalent PFP criteria were low activity (30.5%) and exhaustion (29.8%). Associations were strongest for executive function, where frailty added predictive value beyond its criteria (excess effects of cognitive vulnerability ranging from -0.38SD (SE-0.05) for slowness to -0.47SD (SE=0.06) for shrinking). Slowness was a strong predictor of cognitive change in both frail and non-frail participants, especially for executive function (frail: Cohen’s d per year=-0.16, SE= 0.02; non-frail: Cohen’s d per year=-0.15, SE= 0.02). PFP is an important measure of frailty that adds predictive value beyond its criteria, especially for cognitive levels. Additionally, gait speed remains an important predictor of change in executive function. These results suggest that frailty’s contribution to cognitive performance amounts to more than the sum of its component parts.

